# Molecular docking analysis of phytochemicals from ethanolic extract of crescentia cujete with the auto inhibited parkin catalytic domain

**DOI:** 10.6026/97320630016189

**Published:** 2020-02-29

**Authors:** P Anitha, Praveen K Kumar, P Shanmughavel, TH Nazeema, G Lalitha

**Affiliations:** 1 Department of Biochemistry, Rathnavel Subramaniam College of Arts and Science, Coimbatore, Tamilnadu,India; 2 Department of Bioinformatics, Bharathiar University, Coimbatore, Tamilnadu,India; 3 Michael Job College of Arts and Science for Women, Coimbatore,Tamilnadu, India

**Keywords:** Parkinson disease, Insilico docking, Crescentia cujete, autoinhibited Parkin catalytic domain

## Abstract

The autoinhibited Parkin catalytic domain (PDB ID: 4BM9) receptor has been described to have a role in the ubiquitination of α-syn in Parkinson's disease. Therefore, it is of interest to discuss the molecular docking analysis data of phytochemicals from ethanolic extract of Crescentia cujete with the auto inhibited Parkin catalytic domain. We report the docking features of the phytochemical named 1, 2-Ethanediamine, N-(2-aminoethyl) with the target protein for further consideration towards the design and development of anti-Parkinson agents.

## Background

Neurodegeneration is a process, which involves neuropathological condition, and brain aging due to lack of physical movement and mental relaxations lead to all the stress related disorders [1]. The most second common neurodegenerative disorder is Parkinson disease, which is characterized by four main symptoms slowness of movement, muscle rigor, involuntary and not controllable shaking and loss of postural balance along with some secondary manifestations like a decline in memory, language, problem-solving and other thinking skills that affect a person's ability to perform everyday activities, soft speech and difficulty in swallowing due to uncoordinated movements of mouth with the throat [2]. It is characterized by the loss of pigmented dopaminergic neurons in the substantia nigral pars compacta (SNpc) of the midbrain and the presence of lewy bodies and occurs due to inhibition of mitochondrial complex-1 different mechanisms of cell damage like excitotoxicity, calcium homeostasis, apoptosis, protein aggregation and interaction between genetic and environmental factors [3,4].

Several research studies have been identified the autoinhibited Parkin catalytic domain (PDB ID: 4BM9) is an E3 ubiquitin (Ub) ligase found in Homo sapiens [5]. Mutations in parkin lead to autosomal recessive Parkinson's disease [6]. In addition, it is inactivated due to nitrosative stress [7, 8], dopaminergic stress [9] and oxidative stress [10,11], which were key pathogenic processes in sporadic PD. Thus, loss of parkin E3-ligase activity may not only play a role in autosomal recessive PD, but also sporadic PD. The loss of parkin E3 ligase activity leads to the accumulation of Aminoacyl- tRNA synthetase interacting multifunctional protein type 2 (AIMP2) and far upstream element (FUSE)-binding protein 1 (FBP1) which causes neurodegenerative disorders. Thus, neuropathologic studies of patients with parkin mutations, there is a selective loss of dopaminergic neurons of the substantia nigra and loss of noradrenergic neurons in the locus coeruleus with accompanying gliosis [12].

The available drugs are levodopa, carbidopa, apomorphine, amantadine, orphenadrine, benzhexol, benztropine, selegiline, pergola and many more. These drugs are effectively reversing the symptoms of Parkinson and improve the level of dopamine. The greatest disadvantage at present is available potent synthetic drugs lies in their adverse effects like constipation, ulcer, respiratory depression, hypertension, toxicity and reappearance of symptoms after discontinuation [13]. To overcome the adverse effects, a safe and effective alternate should be developed, as phytotherapeutic agents having neuroprotective effects might be necessary with minimal or null toxicity are formulated in PD treatment. The medicinal plants contain several chemical components of therapeutic value so they can be used as drugs or formulations to treat neurodegenerative diseases.

Crescentia cujeteis a small tree belongs to the family Binoniacea, which grows about 6–10m tall with a wide crown and long branches covered with clusters of tripinnate leaves and gourd-like fruit and these branches are arranged as simple elliptical leaves clustered at the anode. C.cujete leaves are used to treat cold, bronchitis, cough, asthma, urethritis and also to cure haematomas and tumours [14]. The leaves of C.cujete plants have high potential for development as a natural antioxidant and used as a central nervous system depressant in traditional medicine [15]. Therefore, it is of interest to discuss the molecular docking analysis data of phytochemicals from ethanolic extract of Crescentia cujete with the auto inhibited parkin catalytic domain.

## Methods:

### Plant collection:

The leaves of Crescentia cujete were collected from different localities of Coimbatore District and authenticated by the Botanical Survey of India (BSI), Southern Regional Centre, Tamilnadu Agricultural University campus, Coimbatore. A voucher specimen (No: BSI/SRC/5/23/2017/Tech 2021) has been deposited at the Herbarium of the Botany department.

### Preparation of plant extracts:

The leaves were cleaned and shade dried for 7 days, then grounded well to a fine powder. About 600 g of dry powder was extracted with ethanol (80%) at 70°C by continuous hot percolation using soxhlet apparatus. The extraction was continued for 2 days. The ethanolic extract was then filtered and kept in a hot air oven at 40°C for 1day to evaporate the ethanol from it. A greenish brown residue was obtained.

### Gas chromatography-mass spectrometry (GC-MS) analysis:

Ethanolic extract of Crescentiacujetewas lyophilized and the sample was subjected to GC-MS equipment Thermo MS DSQII (Thermo Fisher Scientific, USA) for the analysis. The equipment has a DB 35 - MS Capillary Standard non-polar column with dimensions of 30 mm x 0.25 mm ID x 0.25 µm films. The carrier gas used is Helium with at a low of 1.0 ml/min. The injector was operated at 250°C and the oven temperature was programmed as follows: 60°C for 15 min, then gradually increased to 280°C at 3 min. The identification of components was based on Willey and NIST libraries as well as the comparison of their retention indices [16]. Identification of Active site residues Active site residues for the protein structure 4bm9 was identified by using CASTp server [17]. The residues are SER145, LEU162, ARG163, VAL164, ALA172, THR173, LEU174, THR175, LEU176, THR177, GLU178, GLY179, HIE227, GLU426, LYS427, ASN428, LYS431, HIS433, MET434, LYS435, TRP453, ARG455, MET458, GLY459, TRP462, and VAL465 respectively.

### Toxicity Prediction:

ADMET properties (a total of 23 molecular descriptors) were calculated by using the QikProp program in Schrodinger. QikProp generates physically relevant descriptors and overall ADME properties and drug-likeness parameter, which were used to assess the druggability of the compounds as shown in (Table 1) [18].

### Molecular Docking:

Molecular docking is a process to predict the binding sites of the molecules. Maestro modules [19] in Schrodinger suite were used for docking studies. Glide module [20] were used to predict the interaction of the receptor (PDB ID: 4BM9) with Crescentia cujete derivatives. Docking calculations can be carried out by using SP (Standard Precision) and XP (Extra Precision) modes. In this study XP (Extra Precision) mode were used to predict the binding affinity with OPLS-2005 force field. The molecular docking interactions between the C. cujete with the receptor 4BM9 were described in the (Table 2).

### Preparation of Protein:

**Figure 1:**
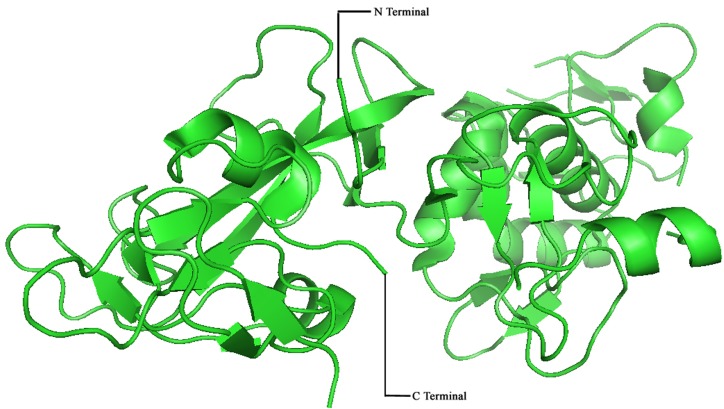
3D structure of the autoinhibited Parkin catalytic domain (PDB ID: 4BM9) Source: RSCB Protein Data Bank, 2018. https://www.rcsb.org/structure/4BM9

The crystal structure of the autoinhibited Parkin catalytic domain was downloaded from Protein Data Bank (PDB) with the PDB ID: 4BM9. The protein preparation was carried out using the protein preparation wizard of Schrodinger Suite 2018. In general, the protein preparation consists of fixing structures, deleting unwanted chains and waters, fixing hetero groups and finally optimizing the fixed structure. The 3D structure of the autoinhibited Parkin catalytic domain (PDB ID: 4BM9) was shown in (Figure 1).

**Figure 2:**
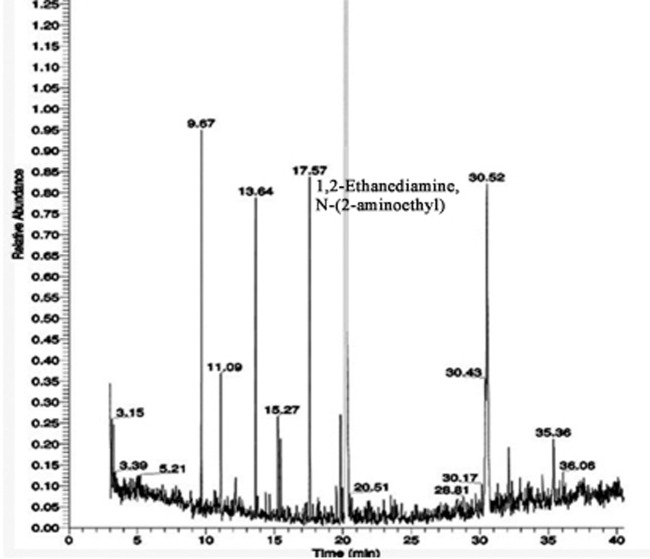
GC-MS chromatogram of ethanolic extract of Crescentia cujete leaves

**Figure 3:**
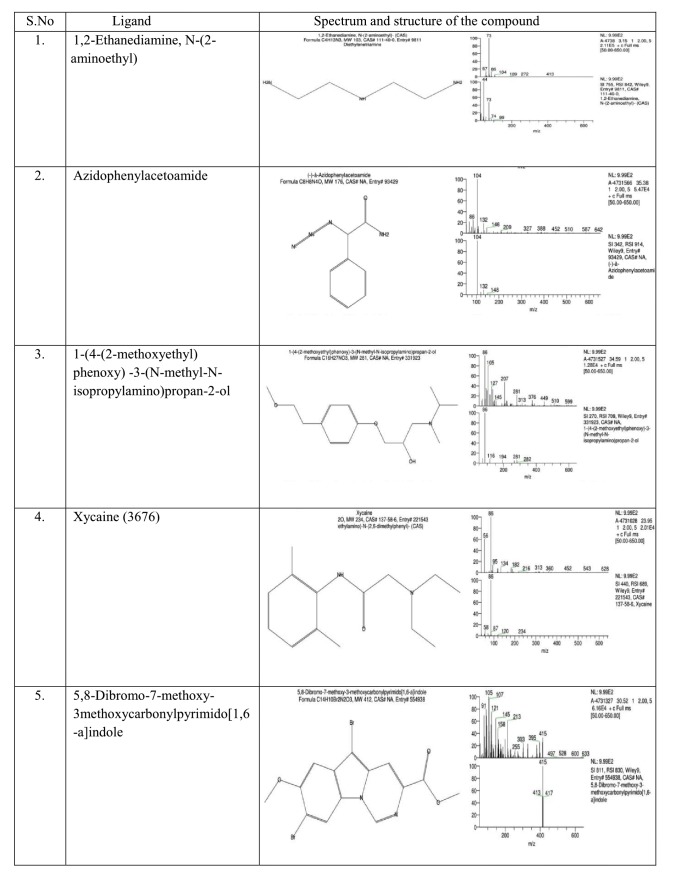
Spectrum and structure of the phyto compound

The structures of the ligands from the results of GCMS analysis were retrieved from PubChem database. Retrieved ligands were prepared by using LigPrep module [21] in Schrodinger Suite 2018. LigPrep includes tautomeric, stereo chemical, tautomeric variations, desalted and corrected for their chiralities, missing hydrogen atoms and flexible filters to generate fully customized ligand libraries that are optimized for computational analysis. The ligands were minimized using OPLS_2005 [22]. Phytochemicals identified from ethanolic extract of Crescentia cujete [23] are given in (Figure 2 and Figure 3).

## Results and discussion:

**Figure 4:**
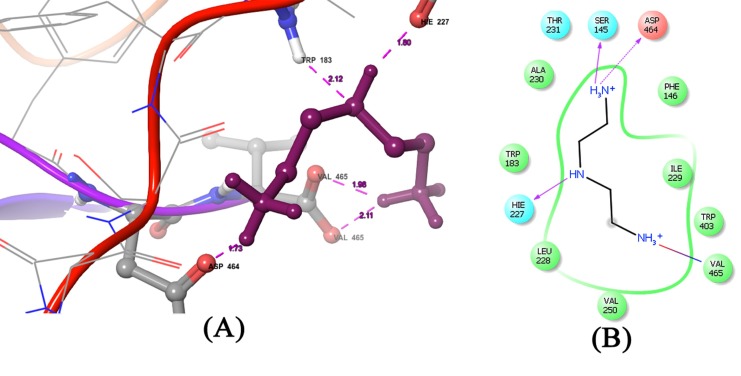
(A) The molecular interaction of 1, 2-Ethanediamine, N-(2-aminoethyl) with the target protein (PDB ID: 4BM9) is shown. Purple dashed lines between the atoms involved indicate hydrogen bonds. (B) A detailed interaction of the compound with the target is illustrated.

Our study is focusing on the inhibition of Parkin catalytic domain. We want to enhance inhibitory activity of autoinhibited parkin catalytic domain, so we are not considering the active form of protein. GlideScore is an empirical scoring function that approximates the ligand binding free energy. It has many terms, including force field (electrostatic, van der Waals) contributions and terms rewarding or penalizing interactions known to influence ligand binding. It has been optimized for docking accuracy, database enrichment, and binding affinity prediction and be used to rank poses of different ligands, for example in virtual screening. As it simulates a binding free energy, more negative values represent tighter binders. The Crescentia cujete compounds that were used for the In-silico study are listed (Figure 3). Molecular interactions of the ligands to 4bm9 were ranked based on various parameters such as Gscore, DockScore and Hbonds. From the docking results, six phytocompounds from Crescentia cujete showed better interaction with 4bm9 (Table 2). Docking Analysis of phytocompounds identified from ethanolic extract of Crescentia cujete are listed in (Table 3). The lead compound has obtained the highest Gscore of -7.41with hydrogen bonds. The DockScore of lead compound was also found to be -7.37. The 4bm9 and lead ligand complex showed in (Figure 4 A and B) interaction with the oxygen atom of the protein molecule and hydrogen atom of the ligand at the amino acid residues VAL 465, VAL 465, ASP 464,TRP 183, HIE 227 with a bond length of 2.11,1.98, 1.73, 2.12,1.80 for both the atoms. The interaction of lead ligand with 4bm9 suggests that it is enhancing the inhibitory activity of parkin catalytic domain for overcoming the disease and makes it beneficiary for identifying novel lead molecule to cure the disease. The Gscore for rest of the five compounds 2 5- dimethyloxazolidine, Xycaine (3676), 1-(4-(2-methoxyethyl) phenoxy)-3-(N-methyl-N-isopropylamino)propan-2-ol, Azidophenylaceto amide and 5,8-Dibromo-7-methoxy-3methoxycarbonylpyrimido [1,6-a]indole were -4.5, -4.96, -5.24, -6.63 and -4.28 respectively.Similarly for the complex 4bm9 with 5,8-Dibromo-7-methoxy-3methoxycarbonylpyrimido[1,6-a]indole, the interacting atom in the protein was found to be oxygen (two atoms) and that of the ligand molecule was Hydrogen (two atoms) and the interacting residues were LYS 299, HIE 227 with the bond length of 2.08, 2.08 respectively. The complex 4bm9 with Azidophenylacetoamide showed interaction with three amino acid residues LEU 228, VAL 465 and HIE227 and the bond lengths were 2.14, 1.50 and 1.85 respectively. The interacting atom of protein was oxygen (three atoms) and that of ligand molecule was Hydrogen (three atoms). 4bm9 and 1-(4-(2-methoxyethyl) phenoxy) showed interaction with three amino acid residues GLN 252, VAL 465 and GLN 252 with the bond lengths 1.94, 1.74 and 2.25 where the interacting atoms were 3 oxygens in protein and 3 hydrogens in the ligand. Two interacting residues at the VAL 465 and GLN 252 were found with the protein complexed with Xycaine. The interacting atom in protein was oxygen (two atoms) and that of the ligand here is also hydrogen (two atoms) and the bond lengths were 1.59 and 2.09 respectively. In 4bm9 and 2, 5- dimethyloxazolidine complex, two oxygen atoms in the protein and two Hydrogen atoms in the ligand showed interaction with the amino acid VAL 465 with the bond length 2.08 for both the atoms.

## Conclusions:

We report the docking features of the phytochemical named 1,2-Ethanediamine, N-(2-aminoethyl) with the target protein for further consideration towards the design and development of anti-parkinson agents. The lead compound satisfying the Lipinski rule of 5 and ADMET properties, to be act as a drug.

## Figures and Tables

**Table 1:** ADMET properties of Crescentia cujete phytocompounds

Molecule	mol_MW	Donor HB	Accpt HB	QP logS	QPlog BB	Human oral absorption
1,2-Ethanediamine, N-(2-aminoethyl)	103.167	5	3.5	2	-0.18	2
(3S)-(3-2H1)-2,2-Dimethylcyclobutyl acet	142.197	0	2	-2.231	0.126	3
1-(4-(2-methoxyethyl) phenoxy	281.394	1	6.15	-2.297	-0.078	3
1-Propyl-1-cyclohexanol	142.241	1	0.75	-2.444	0.16	3
1,3-Dihydro-1-ethylbenzo(c) thiophene 2	196.264	0	4	-1.593	-0.169	3
2-(-N,N-Di-isopro pylamino methyl) -1-methy	194.319	0	2	-2.019	0.717	3
2-Methoxycarbonyl	334.368	0	7.4	-3.466	-0.546	3
2-tert-Butyl-4-trifluoromethyl -1-methyli	206.21	0	1.5	-3.753	0.771	3
2,5- dimethyloxazolidine	101.148	1	3.2	0.566	0.739	3
3-(Phenylethyl) tetrahydrofuran-2-one	190.241	0	3	-1.917	0.036	3
4-n-Butylbenzopyran-4-ol	206.284	1	1.5	-3.348	0.059	3
5,8-Dibromo-7-methoxy-3-methoxycarbonylp	414.053	0	3.75	-5.045	0	3
Anthracene	290.447	0	0	-9.1	0.957	1
Buty l 2-nitropropanoate	175.184	1	2	-2.198	-0.861	3
Eicosane1	364.697	0	0	-15.525	2.047	1
Ethy l1,2, 3,4,5,6,7,8-octahydro-8-oxo-1-n	222.283	0	4	-2.879	-0.101	3
Hexadecanoic acid	284.481	0	2	-7.326	-1.013	1
Hexanoic acid	130.186	1	2	-1.195	-0.54	3
Indolizine	131.177	0	0	-2.974	0.554	3
Phenol, 5-methyl-2-(1-methylethyl)	150.22	1	0.75	-2.331	0.071	3
t-Butylthiothioacetic acid1	220.387	0	2.5	-3.107	-0.04	3
Tridec-2-en-11-ynedial	206.284	0	4	-3.229	-1.928	3

**Table 2:** Molecular interactions of phytocompounds identified from C.cujete to 4BM9 protein

S.No	Ligand	GScore Kcal/Mol	DockScore Kcal/Mol	Binding Energy Kcal/Mol
1	1,2-Ethanediamine, N-(2-aminoethyl)	-7.41	-7.37	-3.27
2	Azidophenylacetoamide	-6.63	-6.57	-1.93
3	1-(4-(2-methoxyethyl)phenoxy)-3-(N-methyl-N-isopropylamino)propan-2-ol	-5.24	-5.21	-2.07
4	Xycaine (3676)	-4.96	-4.94	-1.43
5	5,8-Dibromo-7-methoxy-3methoxycarbonylpyrimido [1,6-a]indole	-4.28	-4.28	-1.33
6	2 5- dimethyloxazolidine	-4.5	-4.21	-1.45

**Table 3:** Hydrogen bond interactions with the compounds, number of hydrogen bonds formed and the distance between compounds and 4BM9 protein.

S.No	Name of Compound	Interacting residues	Bond Length	No of Hydrogen Bonds
1	1,2-Ethanediamine,N-(2-aminoethyl)	VAL 465, VAL 465, ASP 464,TRP 183, HIE 227	2.11,1.98, 1.73, 2.12,1.80	5
2	Azidophenylacetoamide	LEU 228, VAL 465, HIE 227	2.14,1.50,1.85	3
3	1-(4-(2-methoxyethyl)phenoxy)-3-(N-methyl-N-isopro pylamino) propan-2-ol	GLN 252, VAL 465, GLN 252	1.94, 1.74, 2.25	3
4	Xycaine (3676)	VAL 465, GLN 252	1.59, 2.09	2
5	5,8-Dibromo-7-methoxy-3methoxycarbonylpyrimido [1,6-a]indole	LYS 299, HIE 227	2.08, 2.08	2
6	2 5- dimethyloxazolidine	VAL 465, VAL 465	1.91, 2.21	2
